# Surface topography as a material parameter

**DOI:** 10.1557/s43577-022-00465-5

**Published:** 2023-01-31

**Authors:** Tevis D. B. Jacobs, Lars Pastewka, Guest Editors

**Affiliations:** 1 Department of Mechanical Engineering and Materials Science, University of Pittsburgh, Pittsburgh, USA; 2 Department of Microsystems Engineering, University of Freiburg, Freiburg, Germany; 3 Cluster of Excellence livMatS, Freiburg Center for Interactive Materials and Bioinspired Technologies, University of Freiburg, Freiburg, Germany

**Keywords:** Roughness, Surface topography, Contact mechanics, Friction, Adhesion

## Abstract

**Abstract:**

Materials science is about understanding the relationship between a material’s structure and its properties—in the sphere of mechanical behavior, this includes elastic modulus, yield strength, and other bulk properties. We show in this issue that, analogously, a material’s surface structure governs its surface properties—such as adhesion, friction, and surface stiffness. For bulk materials, microstructure is a critical component of structure; for surfaces, the structure is governed largely by surface topography. The articles in this issue cover the latest understanding of these structure–property connections for surfaces. This includes both the theoretical basis for how properties depend on topography, as well as the latest understanding of how surface topography emerges, how to measure and understand topography-dependent properties, and how to engineer surfaces to improve performance. The present article frames the importance of surface topography and its effect on properties; it also outlines some of the critical knowledge gaps that impede progress toward optimally performing surfaces.

**Graphical abstract:**

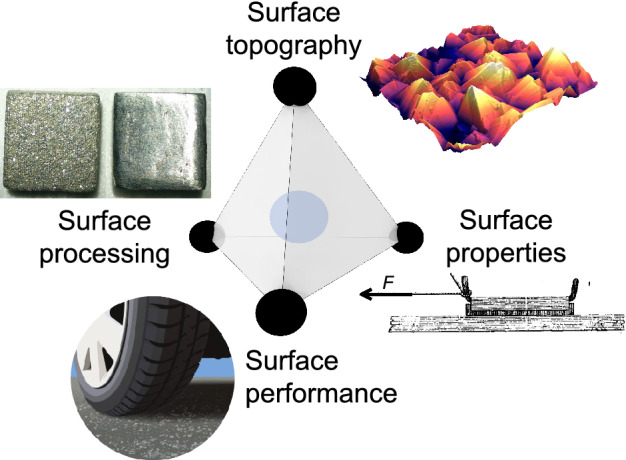

## Introduction: Surface topography as a multiscale material parameter stretching from the size of a component down to the atomic scale

A surface is a planar defect, the outermost layer of a material. Depending on context, it can refer to the outermost atoms, or it can include some depth of near-surface material. The geometry of this surface is a two-dimensional contour, the surface topography. The series of articles in this issue describe how, besides the chemical composition of the material itself, this arbitrarily complex shape of the boundary between a material and the outside world plays a key role in determining the surface properties of that material. Surface topography can control whether paints and coatings flake off of a consumer product, how much energy is wasted in automobile and airplane engines, how quickly a cutting tool wears out, the biocompatibilty of a medical device, and whether a flooring tile will cause slip-and-fall injuries.

We contend that the conventional ways of measuring and describing surface topography impede advancement in this area. Common reference standards (e.g., ISO 4287 or ASTM B46) specify that a surface topography measurement should be separated into three components: large-scale waviness, medium-to-small-scale roughness, and small-scale noise. For example, for the commonly used stylus profilometer, “noise” is typically defined as topography below 2.5 μm of lateral size scale (or wavelength), whereas “waviness” is commonly defined as topography with wavelength larger than 80, 250, or 800 μm (depending on topography and measurement conditions), with everything in between designated as “roughness.” These distinctions serve a practical purpose in a machine shop, where large-scale machining defects can be considered “waviness,” and polishing or other finishing techniques can be used to control “roughness.” However, we contend that these distinctions impede the scientific understanding of topography-dependent properties because the final performance of the part often depends on all scales of topography and does not acknowledge these arbitrary distinctions.

**Figure 1:**
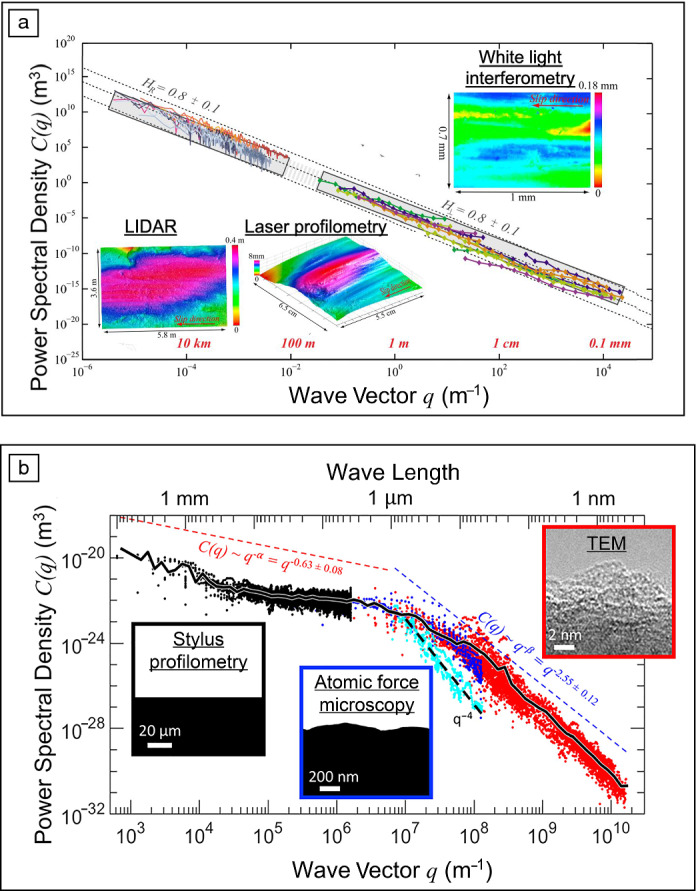
Surface topography can range from kilometers to the atomic scale. (a) The Corona Heights Fault in San Francisco, Calif., provides an example of the multiscale nature of roughness, where roughness features exist over length scales from tens of kilometers down to microns. The surface topography shows self-affine fractal-like scaling, here manifested as a power-law $C\left(q\right)\propto {q}^{-1-2H}$ in the PSD, over this whole range. (b) The topography of ultrananocrystalline diamond, a common wear-resistant coating, deviates from self-affine behavior, but still shows roughness across a wide range of scales from centimeters to Ångströms. TEM, transmission electron microscopy; LIDAR, light detection and ranging. (a) Adapted with permission from Reference 3. © 2012 American Geophysical Union. (b) Adapted with permission from Reference 4. © 2018 American Chemical Society.

We advocate instead to think of the surface topography as a multiscale material parameter, which stretches from the size of a component down to the atomic scale. In an extreme case, the size of the component could be on geologic scales: In this issue, Aghababaei et al.^1^ discuss earthquake faults (**Figure** **1**a), where surface topography has been measured for the same fault on scales stretching from 100 km down to the scale of micrometers with common scale-independent features across all scales. Describing the multiscale nature of surface topography requires spectral analysis, rather than simple scalar metrics. One such example is the power spectral density (PSD), which is a mathematical tool for decomposing a surface into contributions from different spatial frequencies (wave vectors).^2^ An example of the multiscale roughness of an engineering material is given in Figure 1b, where a wear-resistant diamond coating has been characterized from centimeters down to the atomic scale. Taken together, the examples of Figure 1 illustrate that roughness features can exist, and can be statistically characterized, over more than 16 decades in length. All of this multi-scale surface structure can contribute to surface performance.

## Concept: Surface topography is analogous to material microstructure

It is well known within the materials community that microstructure is a critical properties-controlling aspect of a material. Whereas some properties such as elastic modulus can be predicted solely from knowledge of the type of material and its bonding configuration, many other functional properties are governed by the material’s microstructure; these include yield strength, fracture toughness, electrical conductivity, and diffusivity, among others. Specifically, the microstructure comprises the arrangement of crystal grains in the material, their size and orientation, as well as the density and distribution of crystal defects or inclusions or second phases. The performance of a material cannot be truly predicted without knowledge of that material’s microstructure.

Furthermore, microstructural features exist across many orders of length scale. Vacancies, interstitials, and substitutional defects exist at the atomic scale, and can have a critical impact on strength through the interaction of their stress fields with dislocations. Other microstructural features, such as dislocations, twin boundaries, grains and grain boundaries, second phases, and voids or pores can range in size scale from tens or hundreds of nanometers up to the millimeter scale or larger. Describing and optimizing the multiscale dependence of properties on microstructure is a grand challenge in materials science right now, which is captured in priority research directions at the US National Science Foundation (“property prediction across length scales”^5^) and the US Department of Energy (“develop multiscale and multiphysics capabilities [for] multiple length and time scales”^6^).

**Figure 2:**
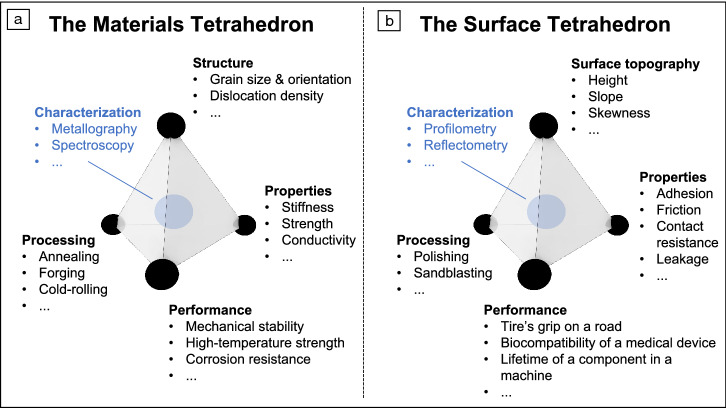
The surface tetrahedron. We propose to think about surfaces in terms of structure–processing–property relationships. Just like the classical materials tetrahedron (a), these relationships can be described schematically by the surface tetrahedron (b).

A similar grand challenge exists for the understanding and optimization of surface topography to control surface properties. Similar to microstructure’s effect on bulk properties, the surface topography has a controlling effect on the performance of the surface. Similar to microstructure, individual topography features can be as small as the size of an atomic defect, or can be as large as the size of the component (see Figure 1). Similar to microstructure, surface topography can be modified through the processing of materials. For these reasons, we propose that the conceptual Materials Tetrahedron (**Figure** **2**a) be complemented by the Surface Tetrahedron (Figure 2b), with surface topography as an integral part.

Unlike microstructure, surface topography often exhibits scale-invariance,^7,8^ which means that measurements performed at one scale can, in many cases, be used to extrapolate structure at other scales. It also makes surface topography especially amenable to multiscale theory and modeling, which has made significant strides in the last two decades as discussed later in the issue. Additionally, a key realization of prior research is that functional properties are often primarily controlled by topographic features over a specific range of scales, where roughness at larger and smaller scales plays little or no role. However, the specific scale that matters is context-dependent.

**Figure 3:**
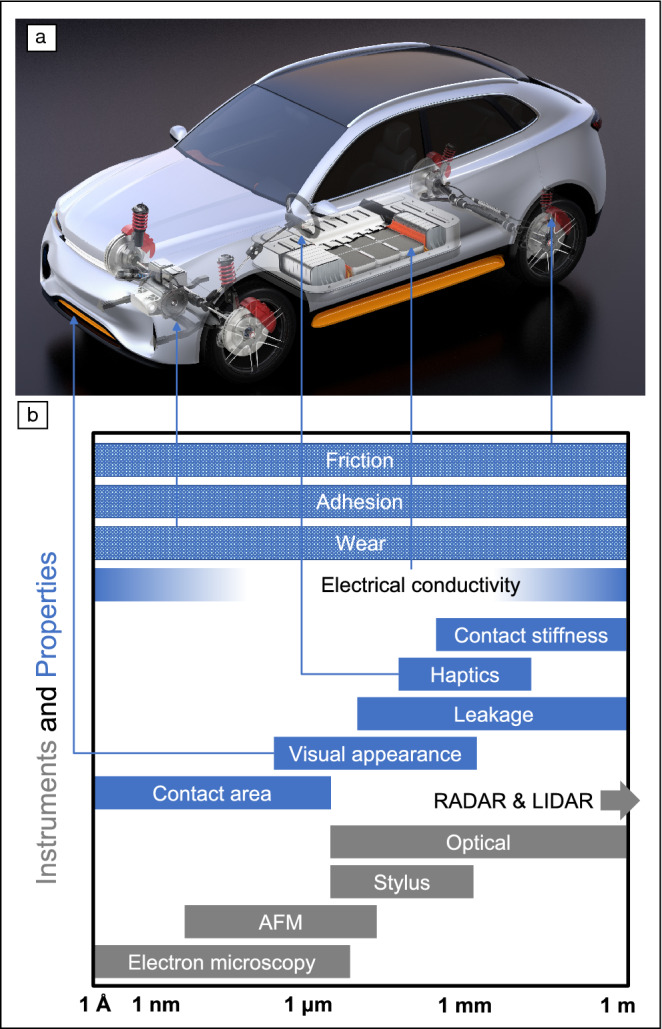
Size scales that control behavior, and techniques that can measure at different sizes. (a) A contemporary electric vehicle has many interfaces whose properties are crucial for its function. (b) Most functional surface properties are governed primarily by certain ranges of size scales. Surface-measurement techniques are also confined to certain length scales, such that multiple methods must be combined for comprehensive topography characterization. AFM, atomic force microscopy; RADAR, radio detection and ranging; LIDAR, light detection and ranging. Image of the electric vehicle from Reference 20, reprinted with permission by L.I.F. Cabrera.

It is possible to broadly categorize some of the key surface properties by the size scale that dominates their performance. We have condensed this knowledge into the conceptual representation of **Figure** **3**. We note that some properties, such as friction, adhesion, and wear cannot be attributed to a single size scale because their dependence on topography depends on the material, and because the scientific understanding is still emerging through active research. But other important properties such as the elastic contact area^9–12^ or contact stiffness^13–17^ have been shown to clearly depend on a limited range of topographic features. Yet other properties depend on multiple distinct scales, such as electric or thermal conductivity that is determined by a combination of a resistance due to constricting the current toward the contacting spots at a rough interface^18^ (that behaves similar to the contact stiffness) and also a contact resistivity^19^ (that is proportional to the total contact area).

We can also map onto the same space the characterization techniques that are able to capture the corresponding scales. For example, certain properties such as the contact stiffness are controlled by large-scale topography and therefore can be understood and controlled by measuring a surface using a stylus or optical profilometer. By contrast, certain other properties such as the contact area (and potentially its derived properties like friction) are controlled by the very smallest-scale topography, and therefore, require techniques with far better resolution. Further refinement is still needed of this understanding of which properties are controlled by topography at which scales, and thus which processing and characterization tools are required for different applications.

## The need for this issue of *MRS Bulletin*: Despite more than 100 years of investigation, we still cannot predict and control topography-dependent properties

It was 110 years ago that Binder^21^ measured that the electrical conductivity of rough surfaces was lower than expected and attributed this to a reduction in contact area due to surface roughness. In the following decades, significant effort went into understanding surface topography and topography-dependent properties. Experimentally, empirical truths were learned such as that the true contact area between rough surfaces increases in direct proportion to the load applied. For characterization, there was a proliferation of tools to measure roughness, from stylus profilometry to white-light interferometry to the atomic force microscope. The theory and modeling of topography-dependent properties has been especially successful. Early models, such as the famous Greenwood and Williamson model,^22^ used oversimplifying assumptions, but accurately captured certain trends in behavior and prompted the scientific community to think quantitatively about roughness. Recent models, especially by Persson,^11,23^ described in this issue,^24^ take account of the multiscale roughness, and result in far more accurate predictions. Also, in recent decades, the atomistic simulation and numerical modeling has made great strides in directly describing and predicting behavior; the progress in this area is summarized by Müser and Nicola^25^ in this issue.

However, despite these advancements, the community as a whole still remains unable to reliably apply these models to real-world surfaces. A useful example of such a failing is provided by a review in the field of bone-tissue engineering.^26^ In this article, the authors discussed the topic of creating scaffolds to induce bone growth and reviewed various aspects of the materials and design and their impact on the growth of bone cells. However, when it came to surface topography, the authors concluded the following: “Overall, the effect of surface roughness (at both the nano- and micron-scales) and grain size on cell adhesion is inconclusive due to contradicting data in the literature.” (p. 8041)

There were simply too many articles showing that cell adhesion increased with roughness (such as Reference 27) and too many articles showing that cell adhesion decreased with roughness (such as Reference 28). The problem is not that the individual studies were performed badly or were inconclusive in isolation; the problem is that the standard practices for measuring and reporting topography give only an incomplete picture of the roughness of a surface.

The community still remains unable to reliably modify surface roughness to control properties. Typical net-shape manufacturing processes yield an uncontrolled, and often quite rough, surface finish. The present solution is then to postprocess a part (e.g., through processes such as polishing, lapping, honing, or grinding). Some surface-modification techniques have been developed (as described in this issue by Costa et al.^29^) to intentionally impart topographic features to the component, yet further work is required to better understand this process and to design specific topographies to achieve certain functional surface properties.

A key impediment to fundamental understanding and control of surface topography is the current practice in topography measurement. The most common practice for measuring topography of manufactured components is to use a stylus or optical profilometer, and to use this to measure a simple roughness parameter such as average deviation from the mean height *Ra*, or root-mean-square deviation from the mean height *Rq*. However, the key limitation of current practice is that typical roughness techniques and metrics capture only a limited range of scales. By filtering out the large and small scales, typical roughness metrics focus primarily on the range of tens to hundreds of microns. This range of sizes will govern only a subset of properties (see Figure 3) and will have little or no predictive power for many other surface properties. Additionally, because of the finite tip radius of a stylus tool and the diffraction limit of light, commonly used techniques do not accurately measure topography below a few microns. Even when atomic force microscopy is used to capture smaller scales, it is rarely combined with large-scale topography for a full description. For these reasons it is, in many cases, impossible to find meaningful correlations between conventional roughness metrics, such as *Ra* or *Rq*, and performance metrics such as adhesion, friction, or surface stiffness.

## Recent advances and future prospects for understanding and controlling surface performance through surface topography

This issue contains five articles targeted at explaining the recent advancements and future directions for the field. The first section contains two articles about the fundamental understanding of topography-dependent properties. Persson^24^ describes his seminal theory of multiscale roughness, and describes a few of the applications for which it has found utility. This theory revolutionized the understanding of topography and pushed other practitioners in the field (theorists, modelers, and even experimentalists) to account for many different size scales of topography. Müser and Nicola^25^ then describe recent advances in the modeling and simulation of the properties of rough surfaces. Unlike analytical theory, which must make simplifying mathematical assumptions about the behavior of the surfaces, these models and theories can consider arbitrarily complex geometries, can include atomistic detail such as the role of dislocations in near-surface plasticity, and solve by “brute force” calculations contact problems that are analytically intractable.

The second section contains three articles about the understanding of real-world surfaces. Aghababaei et al.^1^ address the basic question of how topography emerges on surfaces. This covers both natural surfaces, including an earthquake fault line, as well as human-made surfaces during manufacture and use. Weber et al.^30^ describe experimental observation of adhesion and friction of rough surfaces. Focusing on dry, unlubricated contacts, they show the disparate physics governing the performance of hard and soft materials in contact with rough surfaces. Finally, Costa et al.^29^ describe how surface patterning can be used to intentionally modify adhesion, friction, or heat transfer. The article covers both conventional and additively manufactured components, as well as a variety of environments, including dry, wet, and lubricated.

## Missing links to achieving a comprehensive and predictive understanding of surface topography

Together, the present collection of articles attempts to capture the current state of knowledge in controlling surface performance through surface topography. Each article also contains some insights about the future prospects and critical questions in that particular area. In order to continue advancing this field to the point where we can controllably tailor surface performance, we see that three things are needed:

*First, the roughness-focused community needs to move away from conventional approaches, such as measuring topography using one technique and then computing simple scalar metrics (e.g., Ra); instead moving toward novel approaches, such as measuring topography using many different techniques and then combining their results using multiscale metrics*. So long as simple scalar metrics (any scalar metrics) constitute the most commonly used descriptor, then physics-based predictions will remain impossible. Instead, if multiscale metrics are used (any multiscale metrics), then there is a greater chance of incorporating real physical phenomena and getting closer to fundamental understanding and control.

*Second, materials-science researchers need to begin publishing their topography data (not just computed metrics) along with their scientific publications.* In general, the materials-science community has recognized and embraced the open-data revolution, and it is becoming increasingly common for researchers to publish the raw data collected during their investigations. Indeed, some funding agencies and journals are now requiring it. However, when it comes to topography, this frequently means publishing only metrics, such as *Ra* or the PSD. To achieve significant advances, researchers must more commonly publish their raw topography data, and do so in accordance with the principles of FAIR data,^31^ which are just starting to be embraced in the tribology and surface-roughness communities.^32,33^ One way to publish topography data in an accessible format with a Digital Object Identifier (DOI) is to use the *contact.engineering* platform developed by the present authors.^33^ Regardless of platform, the publication of topography data will reduce the gulf that currently exists between modelers, who have accurate theory/simulations of topography-dependent performance but inadequate access to the topography of real-world surfaces, and experimentalists, who have access to real-world surfaces but typically lack the specialized knowledge and computing resources to implement complex theories or models.

*Third, new metrics are required to describe and report the surface topography adequately*. Although the power spectral density is widely used, including by the present authors, it has its own limitations, including a lack of phase information, ambiguity of implementation, and difficulty of interpreting. Other multiscale metrics have their own advantages and limitations. Also, statistical parameters inherently describe the global statistics of a surface, but fail to capture specific features that could, in some cases, be relevant to performance. Therefore, we hope that the field will continue to find new and better metrics that address these limitations. It is unlikely that some perfect metric will be discovered that is appropriate for all cases; it is far more likely that our understanding will develop and it will turn out that different metrics are particularly useful for different physical situations.

Finally, at the time of this writing, the authors are conducting the Surface Topography Challenge,^34^ in which standard samples are being sent out to any interested research group that focuses on measuring surfaces and their properties. At the completion of the challenge all submitted data will be published for all to see and access. The objectives of this Challenge are in alignment with the needs previously identified: further insight into the strengths and limitations of various techniques for measuring topography; the open publication of the most comprehensive statistical description of a surface ever created; and the release of such a surface so that theorists, simulators, and modelers can apply their calculations to this real-world surface, and also suggest new metrics for describing surface topography.

## Conclusion

In conclusion, we urge the materials-science community to view surface topography as a material property. Using the concept of the surface tetrahedron, the performance of a material’s surface can be understood and controlled analogously to how we routinely modify bulk material parameters. The ultimate goal for the field is to be able to systematically design, impart, and measure surface topography for the rational control of surface properties from adhesion to friction to biocompatibility to electrical and thermal transport across a contacting interface. The recent advances described in the present issue, and ongoing fundamental investigations in the field, are moving the community toward achieving this goal.
